# Various CVD-grown ZnO nanostructures for nanodevices and interdisciplinary applications

**DOI:** 10.3762/bjnano.15.112

**Published:** 2024-11-11

**Authors:** The-Long Phan, Le Viet Cuong, Vu Dinh Lam, Ngoc Toan Dang

**Affiliations:** 1 Faculty of Engineering Physics and Nanotechnology, VNU University of Engineering and Technology, 144 Xuan Thuy, Cau Giay, Hanoi, Vietnam; 2 Graduate University of Science and Technology, Vietnam Academy of Science and Technology, Hanoi, Vietnam; 3 Institute of Research and Development, Duy Tan University, Da Nang 550000, Vietnam; 4 Faculty of Environmental and Natural Sciences, Duy Tan University, Da Nang 550000, Vietnam

**Keywords:** chemical vapour deposition, electron microscopy, Raman and photoluminescent spectra, ZnO nanostructures

## Abstract

This work presents a simple chemical vapour deposition (CVD) method to grow ZnO nanostructures. By annealing Zn powder under atmospheric pressure conditions, we collected nanocrystals with various morphologies, including rods, pencils, sheets, combs, tetrapods, and multilegs. Raman scattering study reveals that the samples are monophasic with a hexagonal structure, and fall into the *P*6_3_*mc* space group. Depending on the morphology and crystal quality, their photoluminescence spectra have only a strong UV emission associated with the exciton radiative recombination, or both UV and defect-related visible emissions with their relative intensity ratio varying with the excitation power density. The obtained results prove that ZnO exhibits many novel nanostructures that can foster the development of next-generation optoelectronic nanodevices and new applications in biological and biomedical fields.

## Introduction

In recent decades, nanomaterials whose diameters are in the range of 1–100 nm have been of intensive interest because they exhibit dimension-dependent intriguing behaviours that are different from their bulk counterparts. These special behaviours come from quantum confinement and surface effects dependent on the surface-to-volume ratio, which directly influences the electronic structure and the crystal structure symmetry. Thus, the study and fabrication of nanomaterials not only aim at exploring novel approaches of quantum physics, but also at realizing new multifunctional electronic/optoelectronic devices, energy storage/generation systems, and renewable energy conversion devices with high performance and low-power consumption [1–3].

In comparison to semiconductors, ZnO has attracted much more attention. This is due to ZnO having outstanding semiconductor behaviours in comparison to other compounds [4–6]. Specifically, its large bandgap energy *E*_g_ ≈ 3.4 eV is comparable to GaN – a typical material for blue-light-emitting diode (LED) technology [7–8]. Also, its exciton binding energy is higher than the thermal energy at 300 K, and it has high-quality optical microcavities [9]. Additionally, it is a transparent semiconductor with significant piezoelectricity [10]. These noble characteristics suggest ZnO to be a potential material in the fabrication of UV/blue/green LEDs, solid-state random lasers, UV-absorption devices, and nanogenerators [9,11–13]. Magnetic ordering can also be established in ZnO lattices upon doping with transition-metal and/or rare-earth elements (known as magnetic semiconductors, DMSs). This is expected to enable the development of next-generation spintronic devices [14] applicable to quantum and neuromorphic computing for artificial intelligence and internet of things [15–17].

Particularly during material fabrication processes, it has been discovered that ZnO exhibits many interesting structures in the nanoscale, such as rods, wires, rings, tubes, helixes, stars, bows, propellers, and cages [18–24]. Together with DMSs, these nanostructures will be beneficial to the development of new ZnO-based materials for photocatalytic [25], biomedical [26], gas sensing [27–28], and flexible electronic/optoelectronic applications [29–30]. They are usually fabricated by chemical vapour deposition (CVD) or solid-vapour phase thermal sublimation [18,23], thermal evaporation [21], hydrothermal method [31–33], and other facile chemical/physical routes [34]. The changes in fabrication and processing conditions will influence the shape and size of ZnO nanostructures.

When using CVD, vapour sources can be Zn powder or a mixture of powdered ZnO and C that are placed in the centre of the tube furnace. They are heated to high temperatures to create Zn vapours, which will be transported by a carrier gas and deposited onto substrates arranged at the downstream/upstream end with a suitable temperature range to form nanostructures [23,35–36]. Our current work uses this simple method to grow ZnO nanostructures. After fabrication, the crystal quality of nanostructures is assessed through Raman scattering (RS) and photoluminescent (PL) measurements at room temperature.

## Experimental

As mentioned above, CVD was utilized to prepare ZnO nanostructures. A commercial Zn powder was used as the starting material (a vapour source) which was loaded in a ceramic bath. Clean Au-coated Si(001) substrates were arranged upstream on an alumina plate that was placed on the bath, nearby the vapour source. This system was placed in the centre of a horizontal quartz tube furnace, see [37] for more detail. The furnace tube was also connected with a gas line and a rotary vacuum pump oil. Before the growth, air was sucked out of the tube by backfilling it with argon (Ar) gas, and then pumped out until the base pressure went to ≈2 × 10^−3^ Torr. After that, the gas mixture of Ar/O_2_ ≈ 4:1 at a flow rate of ≈300 sccm was introduced and used as a transport gas, which ensured the growth condition to be at atmospheric pressure. The growth was executed at a temperature range of *T* = 600–700 °C. After growth for 6–10 h, the CVD system was cooled down to collect white products formed on Si substrates. They were characterized by scanning electron microscopy (SEM, JEOL-6330F) and energy-dispersive X-ray (EDX) spectroscopy. Renishaw’s RS and PL spectrometers operating with laser wavelengths of 488 and 325 nm were also employed to study phonon vibrational and emission spectra, respectively, of typical nanostructures.

## Results and Discussion

**Figure 1:**
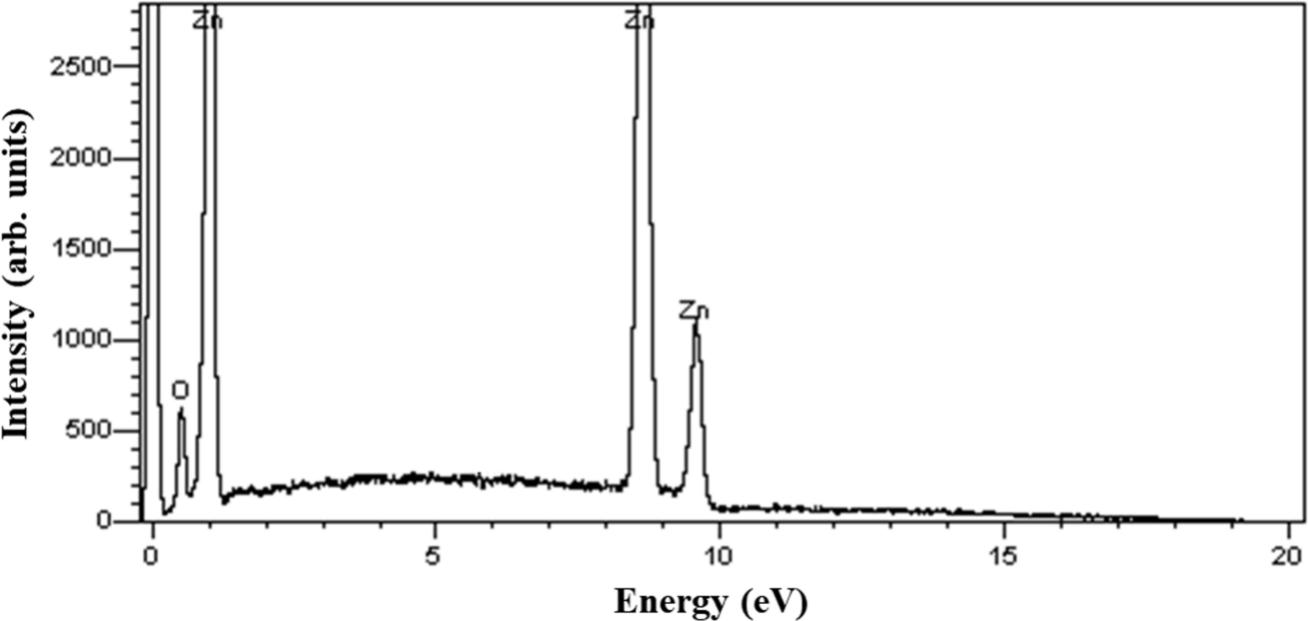
A representative EDX spectrum recorded from ZnO nanorods showing the presence of Zn and O in the product.

We performed multiple experiments of growth of nanostructured ZnO materials by CVD under temperature and gas conditions as aforementioned described. After each growth, we obtained white products deposited on Si substrates. The analysis of their EDX spectra in the energy range of *E* = 0–20 keV shows the presence of Zn and O only, as representatively shown in Figure 1. It means that ZnO crystals were formed, and no impurity was generated during the material fabrication.

Recorded SEM images indicate the products grown on Au-coated Si substrates with various sizes and interesting morphologies. In general, these structures were sensitive to temperature change and were usually formed at temperatures in the range of *T* = 620–650 °C. Hereafter, we shall in turn present nanostructures obtained by CVD.

**Figure 2:**
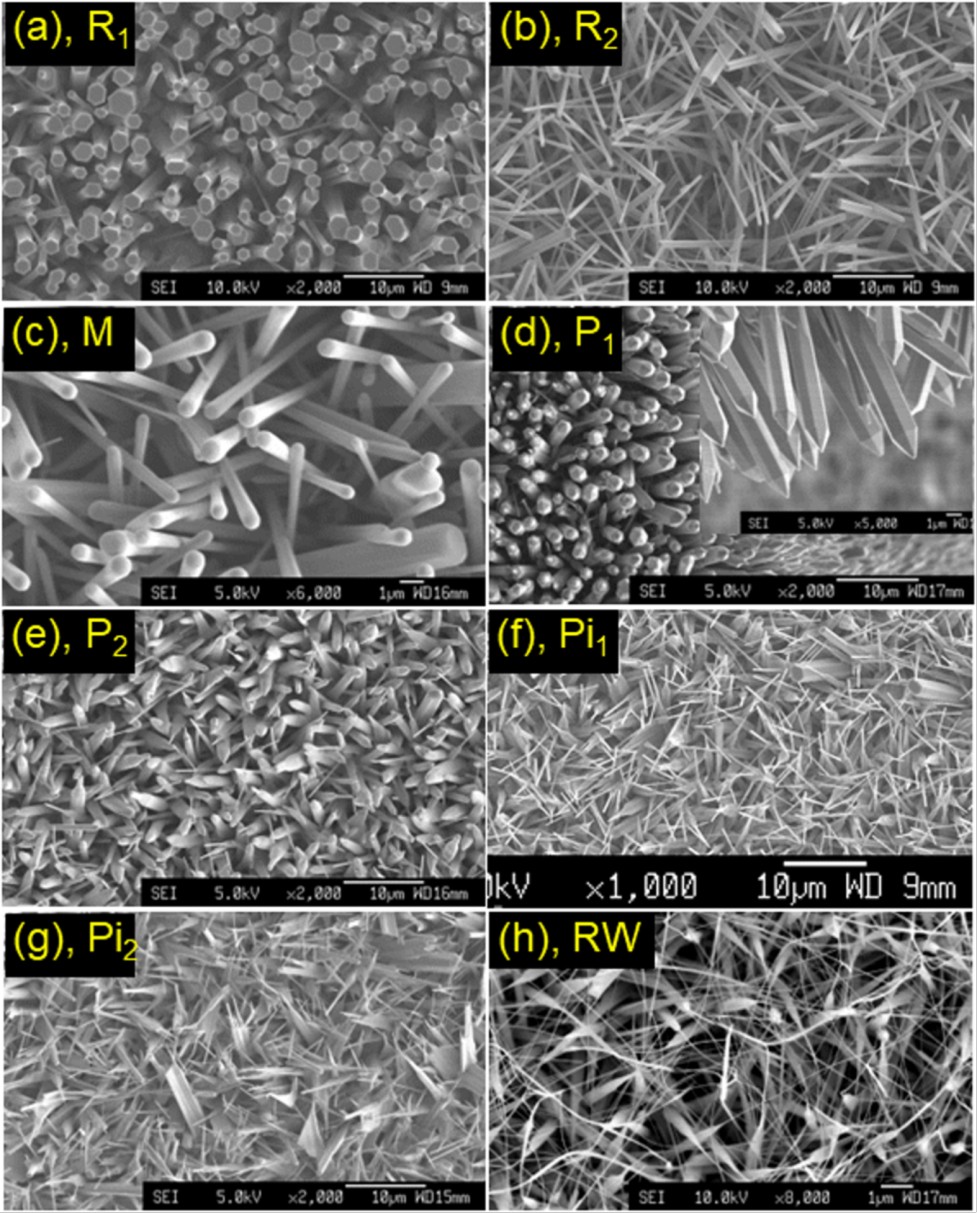
SEM micrographs of some nanostructures: (a, b) rods (R_1_ and R_2_), (c) matches or drumsticks (M), (d, e) pencils (P_1_ and P_2_), (f, g) pins (Pi_1_ and Pi_2_), and (h) rods with wires (RW).

**Figure 3:**
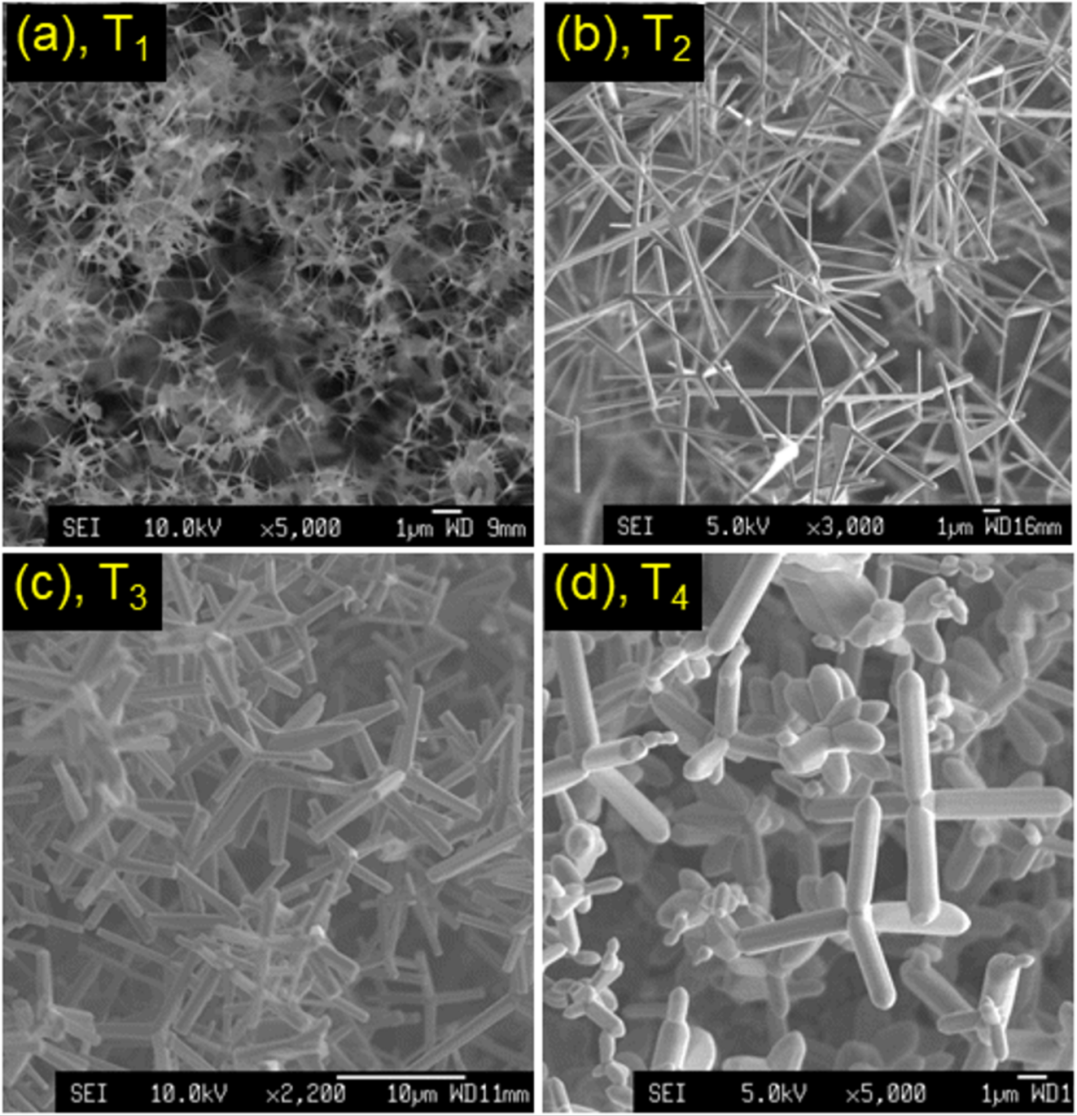
(a–d) SEM pictures of nanoscale tetrapods (T_1_–T_4_) with different morphologies.

Figure 2a–f show SEM micrographs of some 1D-type nanostructures. They were grown at temperatures *T* = 620–630 °C. The first structure is typical of hexagonal-prism-shaped nanorods, named R_1_ and R_2_ in Figure 2a,b. Their diameter can be of several tens to hundreds of nanometres, and their length is about 10–15 μm. The second type of nanostructure has a shape which resembles that of matches or drumsticks, which was mixed with several hexagonal prism-shaped nanorods, named M in Figure 2c. We also collected images of nanopencils, named P_1_ and P_2_ in Figure 3d,e, which have hexagonal prism-shaped bodies with sharp tips similarly to pencils. Their tip lengths can be of several hundreds of manometers [38] or several micrometres – see Figure 2e. Nanopencils with sharp tips can be called nanopins [39], denoted as Pi_1_ in Figure 2f. During the growth, we could collect images of rods with two or three pins, named Pi_2_ as shown in Figure 2g. In this case, rods are usually combined from several single rods. Apart from these structures, we collected images of cylindrical rods with long wires, named RW in Figure 2h. It has been demonstrated that all of these 1D-type structures are single crystals developing along the *c* = ⟨0001⟩ direction [39]. Top and bottom sides/surfaces of these structures are terminated with Zn and O atoms forming positively charged Zn-terminated (0001) and negatively charged O-terminated planes, respectively. Such 1D-type structures have many potential applications in optoelectronic devices [5,39–40], photocatalytic degradation [41], and water splitting for hydrogen technologies [38].

**Figure 4:**
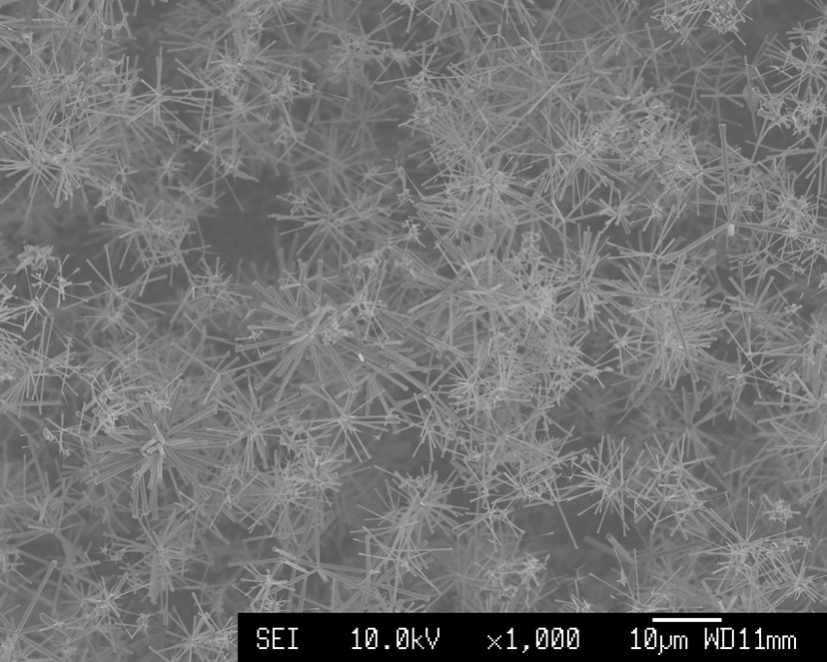
A micrograph showing ZnO multipods grown via CVD.

Figure 3a–d show SEM images of ZnO tetrapods. These various morphologies were grown at approx. 625–650 °C. The first one, named T_1_ in Figure 3a, has needle-like arms of ≈1 μm of length. The dimeter of the arms is less than 50 nm. They converge at the origin with a size of ≈100 nm. For the second morphology, named T_2_ in Figure 3b, the tetrapods have long arms of 8–10 μm, where arms are nanorods with diameters of 200–500 nm. Another tetrapod type shown in Figure 3c, named T_3_, has a morphology similar to that of T_2_ but with shorter arms of ≈5 μm. The other tetrapods shown in Figure 3d, named T_4_, have pestle-like/cylindrical arms with unchanged diameter sizes of ≈800 nm, which were mixed with some multi-arm structures. According to the octa-twin model [42], the central nucleus is an octahedral multi-inversion twin that has eight trigonal pyramidal crystals with three 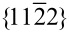 twin planes and a {0001} basal plane [43–44]. Thus, the arms of the tetrapod grow along the ⟨0001⟩ *c*-axis directions, starting from the central nucleus with 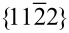 planes. Depending on the growth conditions, not only tetrapods but also octapods and multipods/multilegs (as shown in Figure 4) with different shapes and sizes of arms were also obtained [43,45].

**Figure 5:**
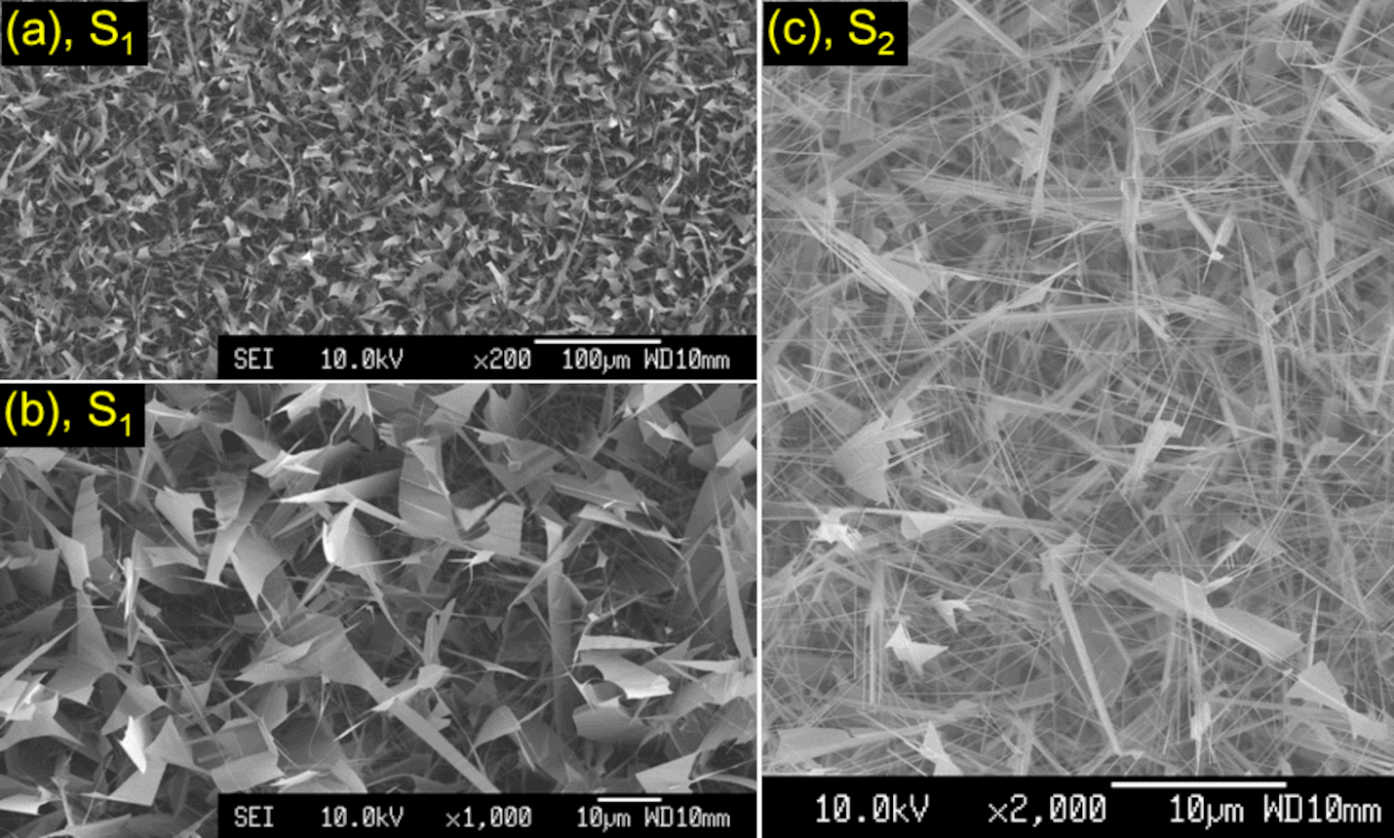
SEM pictures of (a, b) CVD-grown nanosheets, and (c) sheets glued with long nanorods.

Figure 5a,b show SEM micrographs of nanosheets (named S_1_) recorded under different magnifications. They were developed at a temperature *T* ≈ 600 °C. Their length and width are approx. 10–20 and 5–10 μm, respectively. Meanwhile, their thickness is less than 50 nm. Apart from these sheets, we have also collected images of sheets glued with long nanorods that grew along the sheet width, named S_2_ in Figure 5c. Such two-dimensional structures of ZnO are different from those prepared by hydrothermal process [46], radio-frequency magnetron sputtering [47], pulsed laser ablation [48], and electrodeposition methods [49]. They have many application potentials in dye-sensitized solar cells [46], self-powered energy-harvesting devices [47], photocatalysts [48], and turbid lenses [50]. It has been suggested that the preferential growth direction along the length of ZnO sheets is 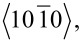 while that along the width is the ⟨0001⟩ direction [51]. This result is similar to the case of comb ribbons found in single-sided tooth combs, as shown below.

**Figure 6:**
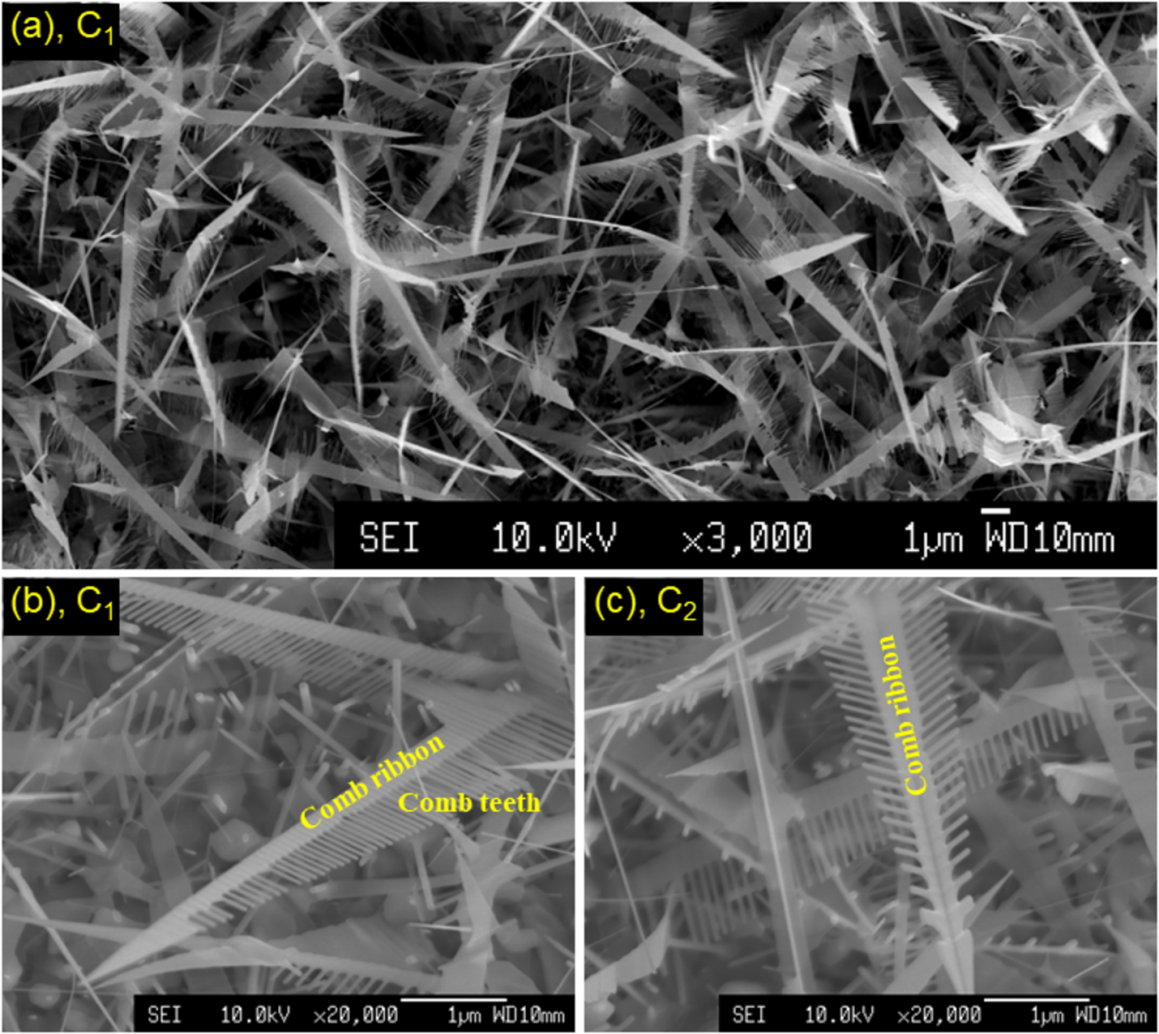
SEM pictures of (a, b) single- and (c) double-sided tooth combs.

We also collected a mixture of single- and double-sided tooth combs that were developed at temperatures of *T* = 630–640 °C. Their length is several to tens of micrometres and the diameter of the teeth is about 50 nm, as shown in Figure 6a–c. As partially shown in [37], electron diffraction analyses indicated all combs to be single crystals. For single-sided tooth combs, named C_1_ in Figure 6a,b, comb ribbon and teeth grew along the 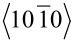 and ⟨0001⟩ directions, respectively. Meanwhile, for double-sided tooth combs, named C_2_ in Figure 6c, comb ribbons are formed from two crystals interfaced at 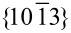 planes, and teeth still grew along the ⟨0001⟩ directions. Besides single- and double-sided tooth combs, there is also a sword-like nanocrystal, see Figure 6c. It is the comb ribbon formed before the teeth are constituted and developed. Combs reported by research groups have various morphologies and sizes, remarkably depending on fabrication conditions [46,52–57]. They are highly sensitive in gas-sensing applications [58–59], and can be used to tune second harmonic polaritons coupling with nanocavity modes and generating polariton cavity modes [60].

The aforementioned results indicate the variety of ZnO nanostructures as well as complex physical mechanisms occurring during their growth. Basically, these structures are formed according to the following processes [61]:


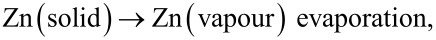



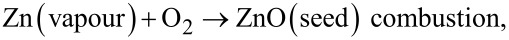



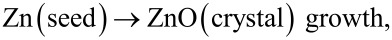


where the oxygen source was from the carrier-gas mixture. For the ZnO hexagonal structure, surface planes {0001}, 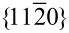, and 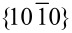 are known as referential and fast growth directions ⟨0001⟩, 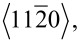 and 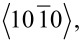 respectively, with the surface energy values in the following order: 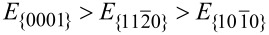 [51]. At temperature and saturated vapour pressure values suitable for referential growth directions, ZnO crystals develop from Zn droplets working as nuclei/seeds, which react with oxygen in order to develop characteristic nanostructures.

**Figure 7:**
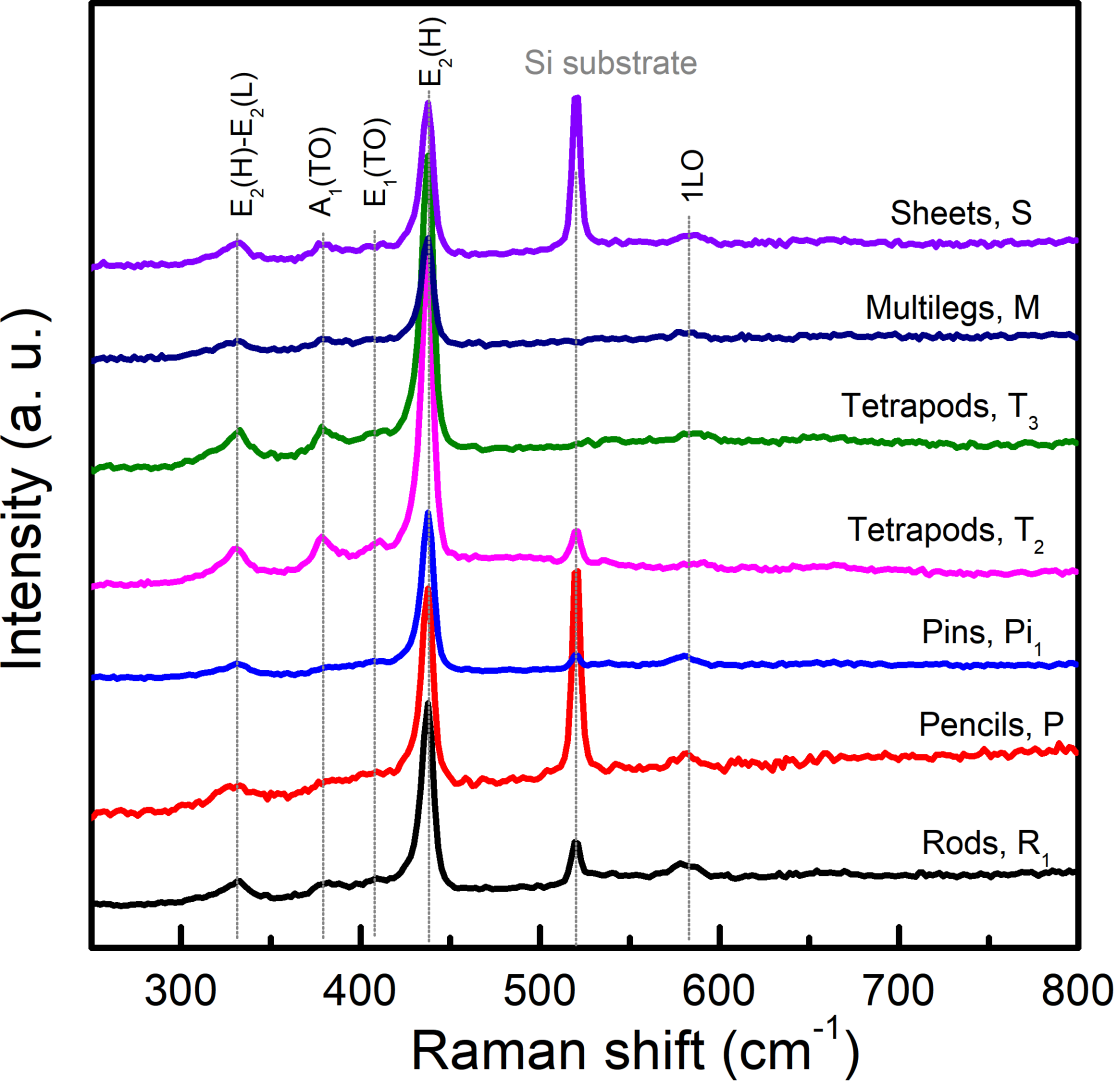
Raman scattering data of some ZnO nanostructures excited at a wavelength of λ = 488 nm. Apart from a Raman mode of Si substrates at ≈520 cm^−1^, all other modes are from ZnO nanostructures.

Following morphological characterizations, we performed RS spectroscopy to check the crystal structure and quality of fabricated ZnO nanostructures. Figure 7 shows RS spectra of typical samples (namely R_1_, P, Pi, T_2_, T_3_, M, and S labelled in the SEM images) recorded in the wavenumber range of 250–800 cm^−1^. All spectra include vibration modes centred at approx. 331, 380, 409, 437, and 582 cm^−1^. They are known as characteristic modes of the *P*6_3_*mc* hexagonal structure of ZnO [17,62]. It has been assigned the strongest mode at ≈437 cm^−1^ to E_2_(L) while the others 331, 380, 409, and 582 cm^−1^ are in turn associated with E_2_(H)-E_2_(L), A_1_(TO), E_1_(TO), and E_1_(LO) processes [62], as labelled in Figure 7. Spectral features recorded from these samples are almost the same. There are no anomalous modes induced by lattice defects, structural disorders, and impurities [17,63–64]. For some nanostructures, however, there are differences in RS intensity of some peaks, which could be due to different crystal orientations and thicknesses. Such results show that our fabricated ZnO nanostructures have a high quality and are single phase in the hexagonal structure.

**Figure 8:**
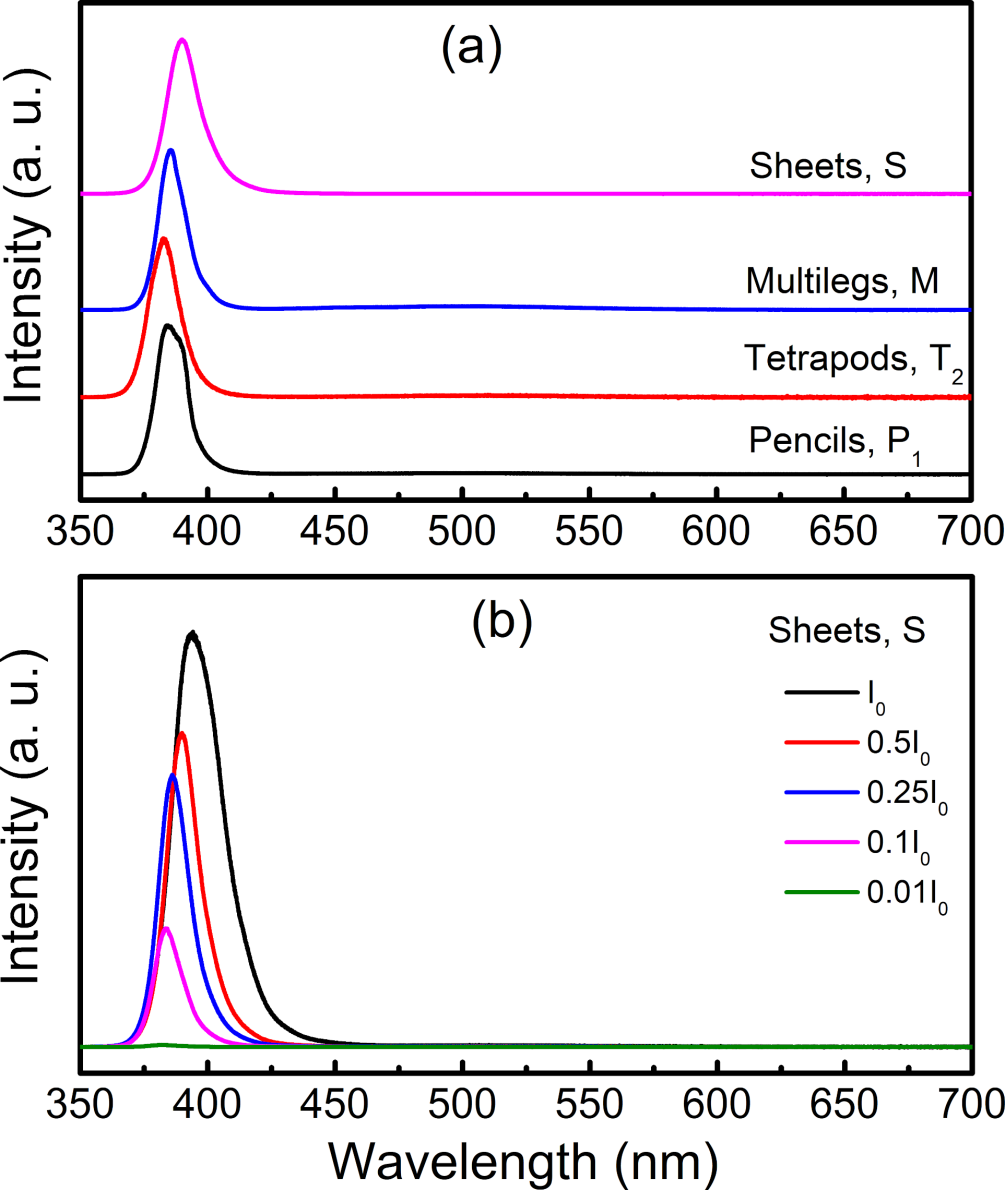
(a) PL spectra of some typical nanostructures (namely P_1_, T_2_, M, and S) showing only a UV emission with laser intensity of 0.1*I*_0_, and (b) spectra of nanosheets (namely S) excited by different laser power densities.

We also measured PL spectra of typical samples under an excitation wavelength of 325 nm, which were measured at wavelengths of 350–700 nm. In this investigation, the maximum excitation laser-power density (*I*_o_) was maintained at ≈170 kW/cm^2^, and filters were utilized to tune the excitation density on experimental samples [65]. Recorded results revealed two main features of PL spectra which can be based on to classify the ZnO nanostructures into two sample groups. The first group gives only a UV emission at approx. 384–390 nm, such as P_1_, T_2_, M, and S shown in Figure 8a. Here, the UV emission is associated with the exciton radiative recombination [66]. An excitation-density increase would remarkably enhance the UV-emission intensity and cause a slight redshift and broadening of the UV line (due to the Coulomb interaction among carriers and exciton–exciton collision processes [67]), see Figure 8b.

**Figure 9:**
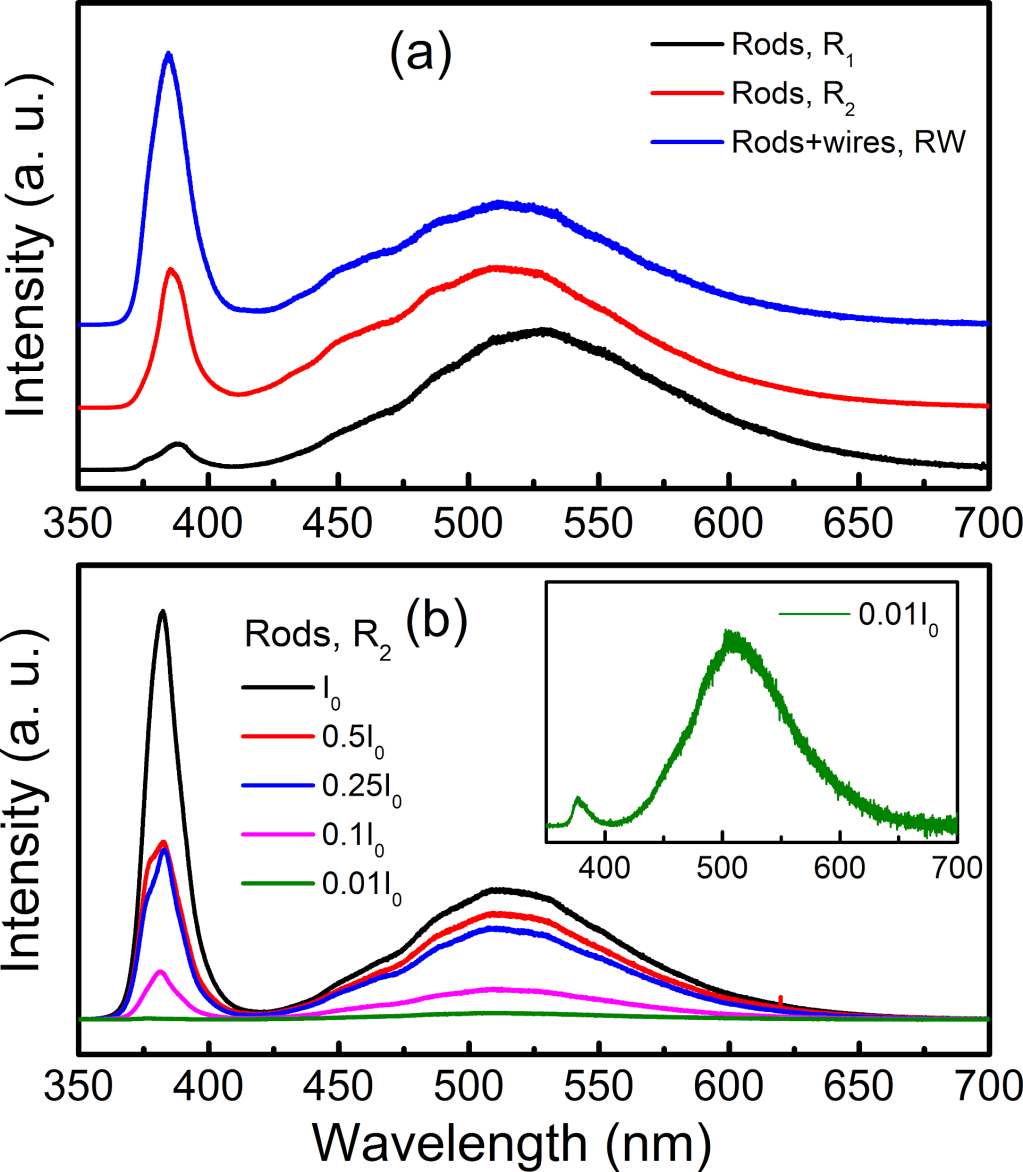
(a) PL spectra of nanorods (R_1_ and R_2_) showing only two UV and visible emissions with laser intensity of 0.1*I*_0_, and (b) spectra of nanorods R_2_ excited by different laser-power densities – the inset is the spectrum for an excitation density of 0.01*I*_0_.

However, the PL spectra of the second group consist of both UV and visible emissions, typically the samples named R_1_, R_2_, and RW, as shown in Figure 9a. The UV emission peaks at ≈384 nm while the visible one peaks at approx. 510–525 nm – corresponding to the green emission that is usually assigned to donor (V_O_)/acceptor (V_Zn_) recombination [68], O_i_ defects [69], or V_O_ defects [70–71]. These defects are intrinsic and nonavoidable during the fabrication of ZnO nanostructures, and are usually dependent on a surface area-to-volume ratio of nanoparticles. Because of this reason, the relative intensity ratio of UV to visible lines can be used to evaluate the ZnO crystal quality [65]. By changing the excitation density, the intensity ratio of these two emissions could also be changed, as illustrated in Figure 9b and its inset for the case of nanorods R_2_. In these samples, after laser excitation, the exciton-related UV emission is partially absorbed by defect centres occupying lower energy levels in the forbidden region. Different from the samples belonging to the first group, defects are insignificant due to their good crystal quality. Their exciton-related UV emission is not massively absorbed by defect centres playing a role as carrier traps, and always give a strong intensity.

## Conclusion

We used CVD to grow ZnO nanostructures, such as rods, pencils, pins, sheets, combs, tetrapods, and multilegs. Their size can be changed in the range from several tens to hundreds of nanometres. These structures usually grow at a temperature range of *T* = 600–650 °C. Raman spectra analysis for characteristic vibration modes proved that the collected ZnO nanostructures are single phase in the *P*6_3_*mc* hexagonal structure. The study of characteristic PL spectra revealed that the fabricated samples could be classified into two groups with different crystal qualities. The first one with a better crystal quality introduced only a UV emission due to the exciton recombination taking place near the band edge. Meanwhile, the other group introduced a PL spectra consisting of both UV and visible/green emissions. Here, the green emission at ≈525 nm is associated with point defects induced during growth. The intensity ratio of these two emissions is strongly dependent on the excitation density of the laser power. We believe that the successful fabrication of single-phase ZnO nanostructures with such optical characteristics will be important for the development of electronic/optoelectronic nanodevices. They also have potential applications in biological, biomedical, and environmental fields.

## Data Availability

Data generated and analyzed during this study is available from the corresponding author upon reasonable request.
